# Levels of cytokines in the aqueous humor guided treatment of refractory macular edema in adult-onset coats’ disease

**DOI:** 10.1186/s12886-020-01474-1

**Published:** 2020-06-30

**Authors:** Yewei Wang, Hua Fan, Ke Gao, Wei He, Yong Tao

**Affiliations:** 1 Dalian He Eye Hospital, Dalian, China; 2 Shanghai Aier Eye Hospital, Shanghai, China; 3 Department of Ophthalmology, He Eye Hospital, He University, No.128, North Huanghe Street, Shenyang, 110034 China; 4 Department of Ophthalmology, Beijing Chaoyang Hospital, Capital Medical University, No. 8, South Road of Worker’s Stadium, Chaoyang District, Beijing, 100020 China

**Keywords:** Macular edema, Adult-onset coats’ disease, Case report

## Abstract

**Background:**

Two cases with refractory macular edema secondary to adult-onset Coats’ disease underwent unsatisfactory treatment by intravitreal injections of anti-vascular endothelial growth factor (VEGF) drugs and retinal photocoagulation.

**Case presentation:**

The authors highlight the guiding effect of the measurement of cytokines in the aqueous humor for the treatment of adult-onset Coats’ disease with refractory macular edema. In the two cases, typical Coats’ disease changes, including telangiectasis, subretinal exudation and macular edema were observed. Initial treatment consisted of intravitreal anti-VEGF drugs and retinal laser photocoagulation; however, the response was poor. Then, the aqueous humor was acquired and the cytokine concentrations were measured **(Flow Cytometry Analysis, Beijing Giantmed Medical Diagnostics Lab**). **When the cytokine levels were tested every time there would be quality control, with a fixed concentration of cytokines samples to detect before the results reported.** A low level of VEGF and a high level of inflammatory cytokines were found. Then, treatment was switched to intravitreal injection of dexamethasone implant (Ozurdex®) (Allergan, Inc., Irvine, Calif., USA), which resulted in resolution of the refractory macular edema and improvement of visual acuity in both cases.

**Conclusions:**

For refractory macular edema secondary to adult-onset Coats’ disease, measurement of the levels of VEGF and inflammatory cytokines can help clinic doctors precisely investigate the molecular mechanism of macular edema and thereby find a suitable treatment.

## Background

Coats’ disease is an idiopathic, nonhereditary retinal vascular disorder characterized by retinal telangiectasis, subretinal lipid exudation, macular edema and capillary nonperfusion areas. It is typically seen in childhood; adult onset is less common. Macular edema is one of the major causes of vision impairment related to adult-onset Coats’s disease.

A conclusive etiology of Coats’ disease has not yet been determined. Several studies have reported increased VEGF levels in the aqueous humor of patients with Coats’ disease [1, 2]. Several case reports have shown that intraocular injection of anti-VEGF combined with laser photocoagulation was effective for adult-onset Coats’ disease [3, 4]. However, treatment of adult-onset Coats’ disease has not been sufficiently investigated, and there have been poor refractory macular edema responses to anti-VEGF treatment. In this paper, we report two cases of adult-onset Coats’ disease with a low level of VEGF and a high level of inflammatory cytokines in the aqueous humor that responded more favorably to intravitreal injection of Ozurdex® compared to anti-VEGF drugs.

## Case presentation

### Case 1

**Fig. 1:**
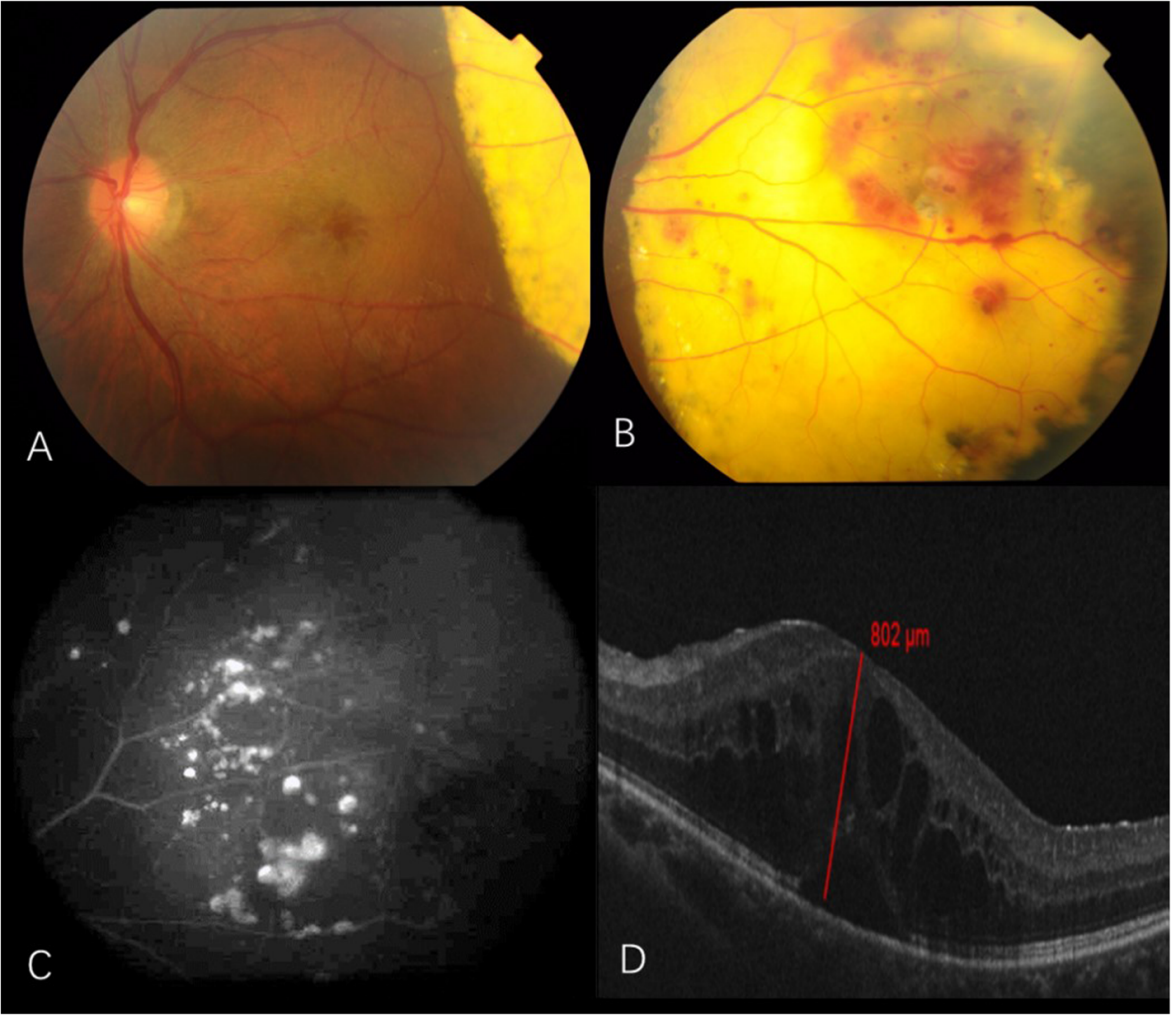
Case 1: **a** and **b** Color photograph showing dense exudation with retinal telangiectasias and aneurysmal dilatations in the temporal retina; **c** Fluorescein angiography showing characteristic light bulb aneurysms and capillary nonperfusion areas seen temporally; **d** Optical coherence tomography showing macular edema with a foveal thickness of 802 μm

**Fig. 2:**
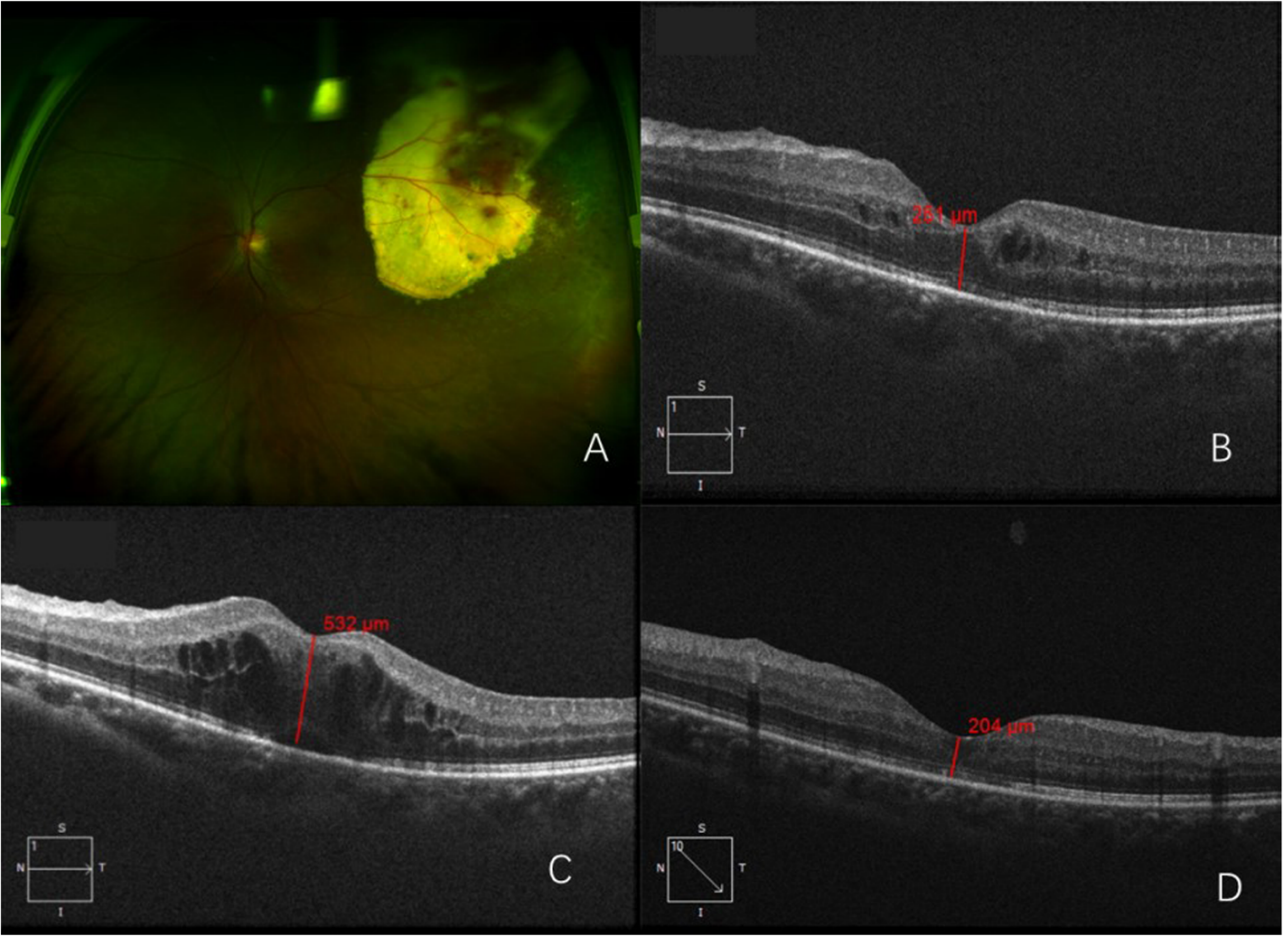
Case 1: **a** Ultra-wide field pseudo-color photograph **(Daytona, P200T, OPTOS PLC, Dunfermiline, UK)** showing exudation with retinal telangiectasias, aneurysmal dilatations and temporally dated laser spots; **b** Optical coherence tomography (OCT) showing decreased macular edema after intraocular Conbercept injection; **c** 4th week after the intravitreal Conbercept injection, OCT showing macular edema recurrence; **d** 6th week after the intravitreal dexamethasone implant injection, OCT shows decreased macular thickness. The retina thickness at the central fovea is 204 μm

A 25-year-old man, without previous medical history, was presented to our eye clinic with decreased visual acuity in the left eye for 3 months. The best corrected visual acuity (BCVA) was 0.3 in the left eye and 1.0 in the right eye, with normal intraocular pressures. A massive intraretinal lipid accumulation with overlying vascular telangiectasis and hemorrhages was observed in the left eye (Figs. 1 and 2a). Fluorescein angiogram (FFA) **(Retinal Camera, TRC-50DX, Topcon Corporation, Tokyo, Japan)** revealed early hyperfluorescence of telangiectatic bulbs in the temporal regions, and hypofluorescence consistent with the subretinal exudates in the left eye. Optical coherence tomography (OCT) **(CirrusHD-OCT 5000, Cail Zeiss Meditec, Inc, Clifornia USA)** showed macular edema in his left eye with a foveal thickness of 802 μm. A diagnosis of adult-onset Coats’ disease was made. Examination of his right eye was unremarkable. Intraocular injection of an anti-VEGF drug (Conbercept) was performed. Before injection, the aqueous humor was removed and the levels of VEGF and inflammatory cytokines were measured. The result showed a normal VEGF level and increased Interleukin (IL) -8 (Table 1). At 1 week after the first injection, the macular edema was alleviated (Fig. 2b), with an increased BCVA of 0.5. However, after 1 month, BCVA decreased again with recurrent macular edema (Fig. 2c). A second injection of anti-VEGF was administrated and again the aqueous humor was acquired before injection. The level of VEGF was low and the level of IL-8 and VACM in the aqueous humor had increased (Table 1). Macular edema remained at this time, and laser coagulation was given at 3 weeks after injection. At 1 month after the second injection, BCVA dropped to 0.3. In consideration of the aqueous humor cytokine concentrations, treatment switched to intravitreal dexamethasone implant (Ozurdex®) (Allergan, Inc., Irvine, Calif., USA). Then the macular edema decreased dramatically (Fig. 2d) and BCVA increased to 0.6 at 2 weeks after injection. No further recurrence of macular edema was noted during 12 weeks of follow-up. Intraocular pressure was elevated at 10 weeks after injection (29 mmHg). After cartiolol hydrochloride eye drops were administrated 2 times a day, the IOP reduced to a normal level. No other adverse effect of the steroid was observed during the course of treatment and follow-up.

**Table 1:** Aqueous humor cytokine concentrations and macular thickness

cytokines	Case 1^1st^	Case 1^2nd^	Case 2	Normal range
VEGF (pg / mL)	26.0	1.9	18.6	0 ~ 40
IL-6 (pg / mL)	36.4	46.0	160.1	1.0 ~ 50
IL-8 (pg / mL)	38.7	35.4	39.4	0 ~ 20
VCAM (pg / mL)	810.5	1626.6	1271.3	200 ~ 1000

*VEGF* Vascular endothelial growth factor; *IL* Interleukin; *VCAM* vascular cell adhesion molecule

### Case 2

**Fig. 3:**
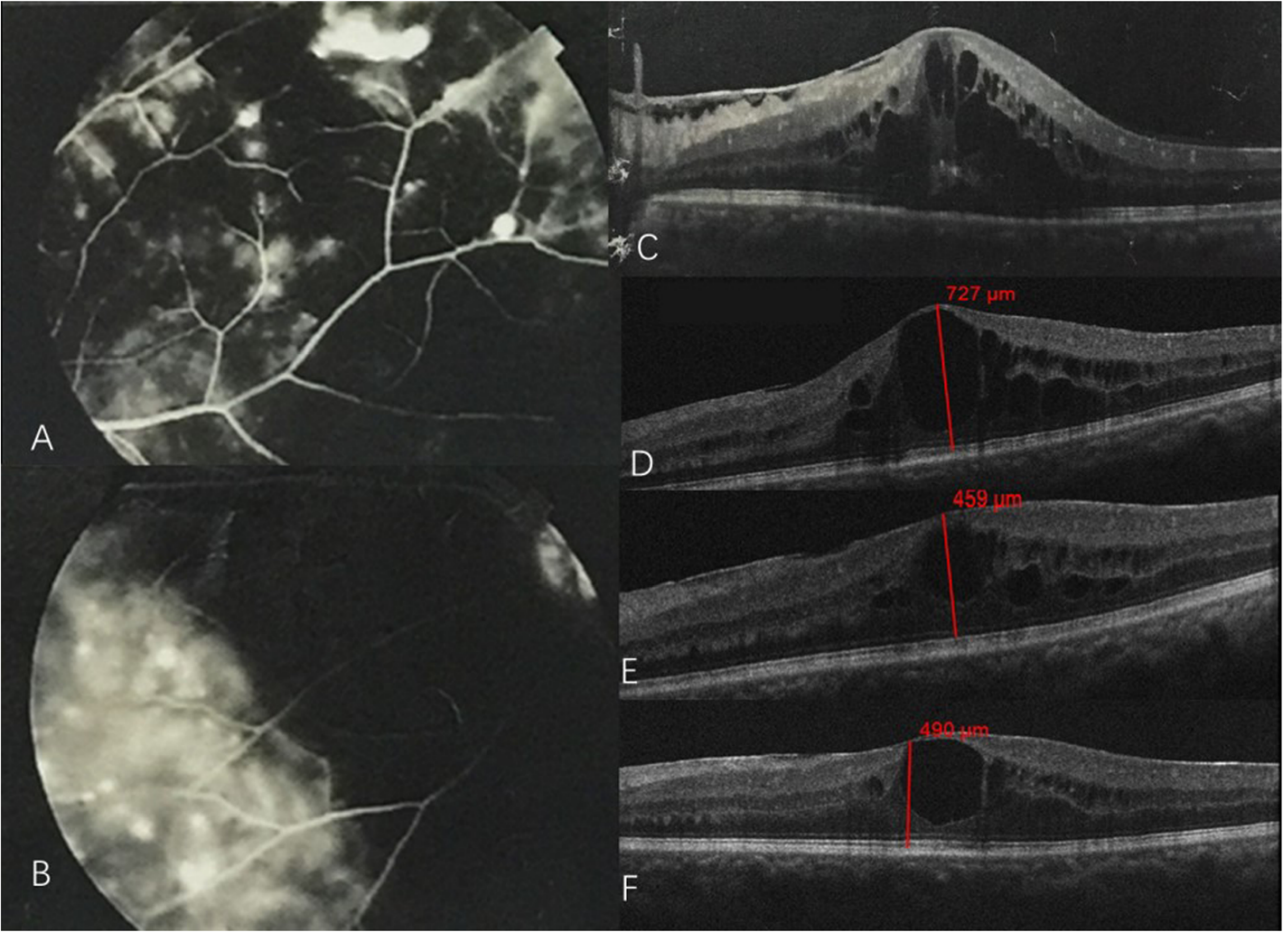
**a** and **b** Fluorescein angiography showing characteristic light bulb aneurysms, capillary nonperfusion areas and leakage from telangiectatic vessels seen temporally and nasally; **c** OCT showing severe macular edema before treatment; (**d**) OCT showing refractory macular edema after 11 treatments of anti-VEGF drug intraocular injection; **e** 1st week after the intravitreal Aflibercept injection, OCT showing decreased macular thickness; **f** 4th week after the intravitreal injection, OCT showing slightly increased macular thickness

**Fig. 4:**
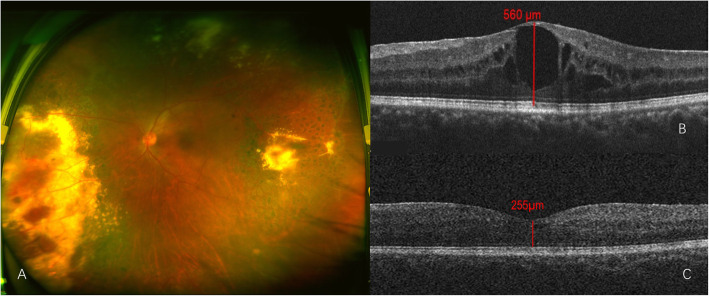
**a** Ultra-wide field pseudo-color photograph showing subretinal lipid exudates in temporal and nasal retina with local vascular telangiectasis and dated laser spots; **b** OCT showing retina thickness at the central fovea is 560 μm before intravitreal dexamethasone implant injection; **c** 12th week after the intravitreal dexamethasone implant injection, OCT showing decreased macular thickness. The retina thickness at the central fovea is 255 μm

A 40-year-old man with 15 months history of macular edema related to Coats’ disease and progressive vison loss in his left eye within 4 weeks was admitted. BCVA was 1.0 in his right eye and 0.2 in his left eye. Subretinal lipid exudates in the temporal and nasal retina with local vascular telangiectasis was observed in the left eye (Fig. 4a). A fluorescein angiogram (FFA) revealed hyperfluorescence of the telangiectatic bulbs, and leaking vessels in the temporal and nasal regions (Fig. 3a, b). OCT showed increased macular thickness, cystoid edema **and mild epiretinal membrane.** (Fig. 3c). A diagnosis of adult-onset Coats’ disease was made. Before he presented to our clinic, he had received 11 intraocular injections of an anti-VEGF drug (Conbercept) and five laser treatments. Initially, he responded well to anti-VEGF drugs; however, there was no improvement after the final injection (Fig. 3d). When he presented to our clinic, he was treated with another anti-VEGF drug via intraocular injection (Aflibercept) and aqueous humor was obtained before the injection. Aqueous humor cytokine concentrations detection showed that VEGF was lower than normal, while VACM, IL-6 and IL-8 were at a very high level (Table 1). At 1 week after injection, macular thickness decreased from 727 μm to 459 μm (Fig. 3d, e); however, it increased to 490 μm again after 2 weeks (Fig. 3f). **Although epiretinal membrane may be also related to the recurrence of macular edema and may influence the cytokine levels.** In consideration of the high inflammatory factors and lower VEGF level, an intraocular steroid (Ozurdex®) injection was administrated. At 10 days after injection, the macular thickness dropped to 255 μm and BCVA increased to 0.5 (Fig. 4b, c). During 3 months of follow-up, visual acuity was stable without any further recurrence of edema.

## Discussion and conclusions

Coats’ disease is classified into 5 stages depending on the severity at presentation: stage 1, telangiectasia only; stage 2, telangiectasia and exudation (2A, extrafoveal exudation; 2B, foveal exudation); stage 3, exudative retinal detachment (3A, subtotal; 3B, total); stage 4, total detachment and secondary glaucoma; and stage 5, advanced end-stage disease [5]. The two cases presented were both stage 2A with extrafoveal exudation; macular edema was the main cause of visual impairment.

While the exact etiology of Coats’ disease remains uncertain, elevated VEGF is found in Coats disease [1], which indicates that VEGF may play an important role in Coats’ disease. Reports have shown that treating the persistent macular edema with anti-VEGF drugs lead to a rapid reduction of the intraretinal liquid as well as improvement of visual acuity [6, 7].

Adult-onset Coats’ disease manifests in a limited area of involvement and seems to advance at a slower rate than it does in children, with most patients reaching a final stable level of visual acuity [8]. Recently, Jing Feng et al. investigated the differences in aqueous concentrations of cytokines in pediatric and adult patients with Coats’ disease; they found that the increasing severity of Coats’ disease is significantly associated with intraocular VEGF concentration in pediatric patients, and that IL-6 may be involved with the inflammatory process in adult patients with Coats’disease [2]. Increased IL-6 concentration was found in one of our cases. Inflammatory cytokines IL-8 and VCAM increased more remarkably than IL-6 in our cases. IL-8 bears the primary responsibility for the recruitment of monocytes and neutrophils, the signature cells of acute inflammatory response [9]. VCAM is crucial for leukocyte recruitment and extravasation during the inflammation process [10]. **MCP-1 was also reported higher in pediatric patients with Coats’ disease than in the control group. Although it is still unknown that MCP-1 level in patients with adult-onset patients, it showed a strongly positive correlation with the extent of retinal exudation** [11]**.** Jun et al. reported an adult patient with Coats’ disease, whose symptom did not improve after intravitreal bevacizumab, while after intravitreal triamcinolone acetonide, the patient showed an obvious improvement in visual acuity and macular edema [12]. Recently, the efficacy of dexamethasone intravitreal implant in the management of adult-onset Coats’ disease has been reported [13]. Since anti-VEGF and anti-inflammatory treatments were both reported to be effective, how to choose the proper treatment remains a question. In our cases, a low VEGF level and a high level of high inflammatory cytokines may explain the poor result of anti-VEGF therapy and the better result of steroid treatment.

The two cases highlight the precise guiding role of aqueous humor cytokine concentrations for the treatment of refractory macular edema in adult-onset Coats’ disease. To the best of our knowledge, little information has been published regarding clinical response and aqueous humor cytokine concentrations in adult-onset Coats’ disease following treatment with anti-VEGF or Ozurdex®.

## Data Availability

Not applicable.

## Abbreviations

VEGF: Vascular endothelial growth factor
IL: Interleukin
VCAM: Vascular cell adhesin molecule
IOP: Intraocular pressure
OCT: Optical coherence tomography
